# Ultrafast Rechargeable Aluminum-Chlorine Batteries Enabled by a Confined Chlorine Conversion Chemistry in Molten Salts

**DOI:** 10.3390/ma18081868

**Published:** 2025-04-18

**Authors:** Junling Huang, Linhan Xu, Yu Wang, Xiaolin Wu, Meng Zhang, Hao Zhang, Xin Tong, Changyuan Guo, Kang Han, Jianwei Li, Jiashen Meng, Xuanpeng Wang

**Affiliations:** 1 Department of Physical Science & Technology, School of Physics and Mechanics, Wuhan University of Technology, Wuhan 430070, China; 2 State Key Laboratory of Advanced Technology for Materials Synthesis and Processing, School of Materials Science and Engineering, Wuhan University of Technology, Wuhan 430070, China; starry_wy@163.com (Y.W.); zhanghao0272@163.com (H.Z.); changyuan551@whut.edu.cn (C.G.);; 3 Institute of Materials Plainification, Liaoning Academy of Materials, Shenyang 110167, China; 4 School of Chemical Engineering, Zhengzhou University, Zhengzhou 450001, China; 5 Zhongyu Feima New Material Technology Innovation Center (Zhengzhou) Co., Ltd., Zhengzhou 450001, China; lijianwei0228@163.com

**Keywords:** chlorine batteries, molten salts, ultrafast, nitrogen-doped structures, aluminum dendrite

## Abstract

Rechargeable metal chloride batteries, with their high discharge voltage and specific capacity, are promising for next-generation sustainable energy storage. However, sluggish solid-to-gas conversion kinetics between solid metal chlorides and gaseous Cl_2_ cause unsatisfactory rate capability and limited cycle life, hindering their further applications. Here we present a rechargeable aluminum-chlorine (Al-Cl_2_) battery that relies on a confined chlorine conversion chemistry in a molten salt electrolyte, exhibiting ultrahigh rate capability and excellent cycling stability. Both experimental analysis and theoretical calculations reveal a reversible solution-to-gas conversion reaction between AlCl_4_^−^ and Cl_2_ in the cathode. The designed nitrogen-doped porous carbon cathode enhances Cl_2_ adsorption, thereby improving the cycling lifespan and coulombic efficiency of the battery. The resulting Al-Cl_2_ battery demonstrates a high discharge plateau of 1.95 V, remarkable rate capability without capacity decay at different rates from 5 to 50 A g^−1^, and good cycling stability with over 1200 cycles at a rate of 10 A g^−1^. Additionally, we implemented a carbon nanofiber membrane on the anode side to mitigate dendrite growth, which further extends the cycle life to 3000 cycles at an ultrahigh rate of 30 A g^−1^. This work provides a new perspective on the advancement of high-rate metal chloride batteries.

## 1. Introduction

The increasing reliance on renewable energy sources, such as wind and solar power, highlights the urgent need for the development of efficient, environmentally friendly, and cost-effective energy storage solutions in contemporary energy systems [1,2]. Lithium-ion batteries are currently the most advanced technology for electrochemical energy storage, known for their high conversion efficiency, excellent cycle stability, and substantial energy density [3,4]. In anticipation of growth in the energy storage market, the production capacity of lithium-ion batteries is continuously being expanded [5]. Nevertheless, the increasing demand for energy storage presents challenges for lithium-ion batteries, including resource scarcity, high costs, and safety concerns [6,7]. Therefore, it is essential to develop robust energy storage systems to mitigate traditional energy depletion and address the intermittency issues associated with renewable energy sources [8,9,10,11,12]. Alternative energy storage technologies, including sodium-ion batteries [13,14], potassium-ion batteries [15,16], aluminum-ion batteries [17,18], and zinc-ion batteries [19,20], have received widespread attention in recent years. Among these technologies, rechargeable aluminum-ion batteries emerge as promising candidates for next-generation energy storage devices due to their high volumetric capacity (8040 mAh cm^−3^), abundant availability in the Earth’s crust (8.2%), and the inherent safety of aluminum anodes [21,22].

The cathode material is a critical component of aluminum-ion batteries, significantly influencing their overall performance [21]. Depending on the type of reaction, cathodes can be categorized into intercalation-type reactions, such as those involving graphite and transition metal chalcogenides [8,23,24], and conversion-type reactions, such as those utilizing sulfur, iodine, and tellurium [25,26,27]. Although graphite is a representative intercalation-type material known for its good cycling stability, its low capacity limits its broader application potential. In contrast, conversion-type cathodes, such as sulfur and iodine, have drawn significant attention due to their higher theoretical energy density compared to intercalation-type cathodes [28]. Chlorine, another halogen similar to iodine, theoretically provides a high capacity of 756 mAh g^−1^ at 1.36 V relative to the standard hydrogen electrode (SHE), with an energy density comparable to that of sulfur [29,30,31,32,33]. With its higher voltage platform and rapid reaction kinetics, chlorine redox processes have the potential to achieve energy densities on par with sulfur while offering superior power densities [34,35]. Furthermore, the abundant presence of chlorine in the Earth’s crust ensures a continuous supply of low-cost and sustainable materials [33,36]. Nevertheless, to date, there have been no reports on the use of Cl₂ cathodes in rechargeable aluminum-ion batteries.

In the 19th century, French scientists developed Zn-Cl_2_ batteries utilizing aqueous electrolytes; however, these batteries encounter challenges such as side reactions (including oxygen evolution) and weak physical adsorption of the cathode material to Cl_2_, leading to low coulombic efficiency [37,38,39,40]. Recently, Zhu et al. successfully developed rechargeable Li/Cl_2_ and Na/Cl_2_ batteries by confining chlorine gas within microporous carbon structures [41]. Rechargeable alkali metal/chlorine batteries present notable advantages, including a high specific capacity of 1200 mAh g^−1^ and a substantial discharge plateau of 3.6 V. However, the formation of solid chlorine salts on the cathode side significantly hinders ion and electron transport, resulting in unsatisfactory rate performance and cycling stability [29,42,43]. Consequently, subsequent research has mainly concentrated on modifying the cathode materials to overcome this challenge, and proposed employing metal–organic frameworks (MOFs) in Li-Cl_2_ batteries to enhance the Cl_2_/LiCl conversion reaction [34]. Zhang et al. [35] applied NH_2_-functionalized COFs in Li-Cl_2_ batteries. Theoretical calculations indicate that the introduction of COF-NH_2_ significantly lowers the decomposition barrier of LiCl, accelerating the kinetics of solid LiCl oxidation to Cl_2_ and enhancing the reversibility of Li-Cl_2_ batteries. Nonetheless, the presence of solid chloride salts continues to impede ionic and electronic transport, while the slow kinetics of solid–gas reactions contribute to unsatisfactory rate performance. Consequently, achieving high coulombic efficiency, favorable rate performance, and excellent cycling stability for the reversible oxidation-reduction reactions involving chlorine remains a substantial challenge.

It is understood that the voltage window of molten salt electrolytes employed in aluminum-ion batteries is constrained by the oxidation reaction of chloroaluminate ions (4AlCl_4_^−^ → 2e^−^ + Cl_2_ + 2Al_2_Cl_7_⁻) [44,45,46]. Drawing inspiration from prior research on the reduction in chlorine gas on carbon within molten salt electrolytes, we propose utilizing the redox reaction between AlCl₄⁻ and Cl_2_ as the cathode reaction, while aluminum deposition and stripping would occur at the anode [47,48]. This approach avoids challenges related to side reactions found in aqueous electrolytes and the issues posed by solid chlorine salts in thionyl chloride electrolytes. Additionally, the rapid reaction kinetics associated with molten salts are expected to result in high coulombic efficiency and excellent power density for the chlorine conversion reactions [49].

**Figure 1:**
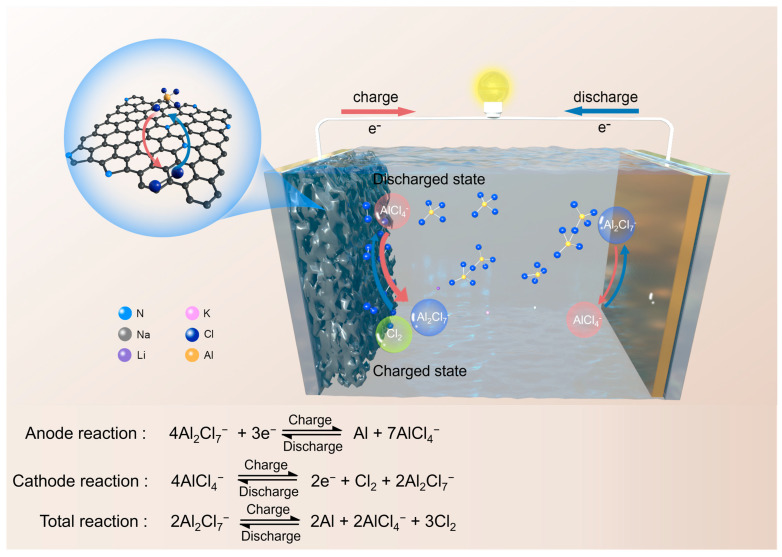
Schematic illustration of the configuration and working mechanism of the molten salt Al-Cl_2_ battery.

Here we develop an ultrafast rechargeable Al-Cl_2_ battery featuring a reversible Cl_2_/AlCl_4_^−^ redox cathode paired with an aluminum anode in the molten salt electrolyte (schematically shown in Figure 1). Ab initio molecular dynamics (AIMD) simulations and density functional theory (DFT) calculations reveal that in traditional ionic liquids, EMI⁺ ions have a strong adsorption effect on chlorine gas (Cl_2_), leading to irreversible capacity loss. Additionally, nuclear magnetic resonance (NMR) also uncovered that during the cycling process, EMI⁺ ions undergo side reactions. In contrast, molten salt electrolytes not only possess high ionic conductivity, low cost, and high safety but also maintain structural stability during cycling. X-ray photoelectron spectroscopy (XPS) and gas chromatography-mass spectrometry (GC-MS) have confirmed a reversible solution-to-gas conversion reaction between AlCl_4_⁻ and Cl_2_ in the cathode, which exhibits faster kinetics compared to solid-gas reactions. Furthermore, density functional theory (DFT) calculations have revealed that nitrogen-doped porous carbon (NPC) enhances the adsorption of Cl_2_, thereby improving the cycle life and coulombic efficiency of the battery. At an operation temperature of 120 °C, the molten salt Al-Cl_2_ battery using the NPC cathode exhibits a high discharge capacity of 243.33 mAh g^−1^, a high discharge plateau of 1.9 V, low polarization voltage of 300 mV, and stable cycling over 1200 cycles at a high rate of 10 A g^−1^. The superior rate capability can be attributed to fast chlorine conversion reaction kinetics in the molten salt electrolyte. To further improve cycling stability, we incorporated carbon nanofiber membranes (CFMs) on the anode side, resulting in uniform aluminum deposition at the anode. At an exceptionally high rate of 30 A g^−1^, the battery demonstrates reversible cycling capabilities that exceed 3000 cycles. This research opens up new horizons for the development of high-performance metal chlorine batteries.

## 2. Materials and Methods

First, Ketjenblack (KB), NaCl–KCl–ZnCl_2_, and 1,4-dicyanobenzene (DCB) are mixed uniformly in a mass ratio of 1:1:1, then the mixture is placed in a dry quartz tube, which is evacuated and sealed. It is heat-treated at a temperature of 300 °C for 48 h (with a heating rate of 5 °C/min and furnace cooling), resulting in KB@COF. KB@COF was transferred into a crucible and then heated at 800 °C for 3 h under argon atmosphere with a heating rate of 5 °C min^−1^ to obtain NPC material.

## 3. Results and Discussion

Due to the lack of Cl_2_ adsorption sites, the released chlorine gas cannot be effectively stored, leading to inevitable chlorine loss and a decrease in coulombic efficiency. Previous studies have demonstrated that high specific surface area and nitrogen-doped structures can better accommodate and adsorb chlorine gas [42,50]. Therefore, we synthesized an NPC cathode with a high specific surface area and nitrogen-doped structure. We uniformly mixed molten salt (ZnCl_2_-KCl-NaCl), 1-4 dicyanobenzene (DCB), and Ketjenblack (KB) and then heated the mixture at 300 °C for 48 h. Under the catalytic action of the Lewis acid ZnCl_2_, DCB undergoes in situ polymerization on the surface of KB to form a covalent organic framework (COF). The resulting product is then further subjected to high-temperature sintering (800 °C, 3 h) to obtain nitrogen-doped porous carbon (NPC) [51]. Our goal was to introduce nitrogen-doped atoms into the material while maintaining the porous structure of KB to enhance its performance in chlorine gas adsorption.

**Figure 2:**
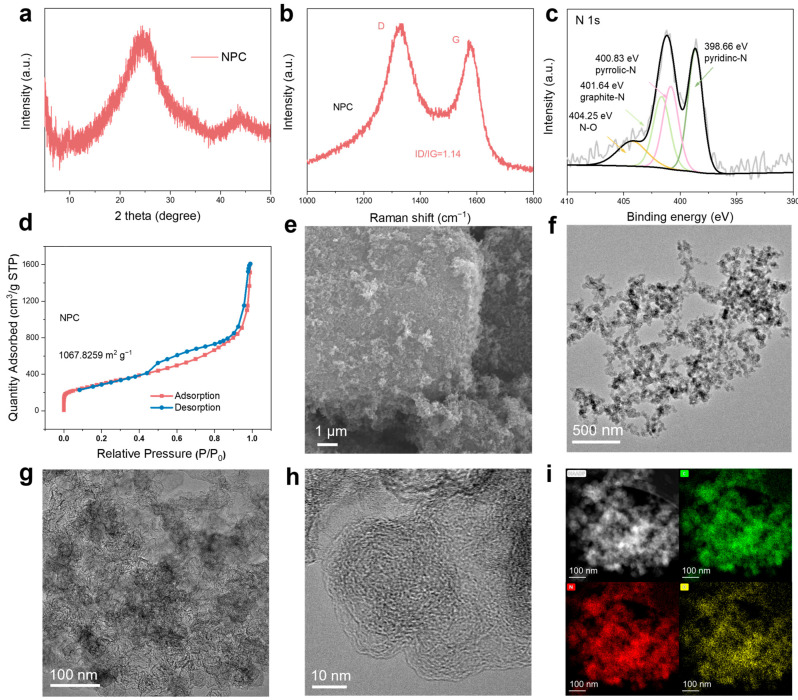
Characterizations of the NPC. (**a**) XRD pattern of the NPC. (**b**) Raman spectroscopy of the NPC. (**c**) N 1s XPS spectra of the NPC. (**d**) Nitrogen adsorption/desorption isotherms of the NPC at 77 K. (**e**) SEM image and (**f**–**h**) TEM images of the NPC. (**i**) The HAADF-STEM image and corresponding EDS mapping images for NPC.

We employed a series of characterizations to analyze the synthesized NPC product. X-ray diffraction (XRD) analysis reveal that the NPC exhibits broad peaks within the 20–30° range, while the original peaks diminished. This suggests that the ordered structure of the COF structure is destroyed at elevated temperatures, resulting in an amorphous structure (Figure 2a and Figure S1a). Raman spectroscopy (Figure 2b and Figure S1b) indicated a comparable level of graphitization for NPC and KB, which supports efficient electron transport in the NPC. X-ray photoelectron spectroscopy (XPS) analysis demonstrated that NPC is primarily composed of carbon (C), nitrogen (N), and oxygen (O) elements (Figure S2a). High-resolution N 1s spectra confirmed the presence of nitrogen in various forms: graphitic nitrogen (401.64 eV; 27.6% of the total N content), pyrrolic nitrogen (400.83 eV; 30.1%), and pyridinic nitrogen (398.66 eV;42.3%) (Figure 2c). The C 1s spectra exhibited a distinct C−N component at 285.49 eV (Figure S2b). Elemental analysis tests further revealed a nitrogen content of 3% (Table S1). The nitrogen adsorption–desorption isotherm of NPC belongs to Type IV isotherm, indicating a pronounced mesoporous structure, with a BET specific surface area (SSA) of 1067.83 m^2^ g^−1^, comparable to that of KB (1380.25 m^2^ g^−1^) (Figure 2d and Figure S3), and pore sizes predominantly below 10 nm. (Figure S4). Scanning electron microscopy (SEM) images revealed that the NPC consists of irregularly shaped porous particles (Figure 2e and Figure S5). High-resolution transmission electron microscopy (HRTEM) revealed the amorphous and porous structure of the nitrogen-doped porous carbon (Figure 2f–h). Energy-dispersive spectroscopy (EDS) analysis confirmed the uniform distribution of C, N, and O elements within the NPC (Figure 2i).

**Figure 3:**
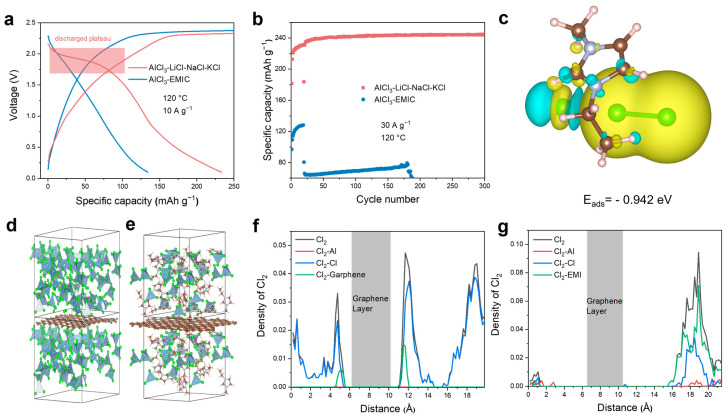
Electrochemical performances and theoretical calculations of the molten salt and ionic liquid Al-Cl_2_ batteries. (**a**) Voltage profiles of Al-Cl_2_ battery assembled with the electrolytes of molten salt and ionic liquid. (**b**) The cycling stability of the Al-Cl_2_ battery assembled with molten salt and ionic liquid with a charge capacity of 250 mAh g^−1^ at a current density of 30 A g^−1^. (**c**) Charge density difference of the Cl_2_ adsorption on EMIC. Snapshots of AIMD simulations for (**d**) ionic liquids and (**e**) molten salt electrolytes. Density profiles of Cl_2_ bonding with different species in (**f**) molten salt and (**g**) ionic liquid.

We chose a quaternary molten salt mixture comprising AlCl_3_, NaCl, KCl, and LiCl as the electrolyte, which previous studies have shown to possess a low melting point and high conductivity [52]. As shown in Figure 3a, the Al-Cl_2_ battery using this molten salt exhibits a distinct chlorine evolution plateau (~2.25 V) and a reduction plateau (~1.95 V) at a current of 10 A g^−1^ and an operation temperature of 120 °C. In contrast, there is no discharge plateau for the ionic liquid electrolyte. Furthermore, we investigated the electrochemical performances of different electrolytes at a current density of 30 A g^−1^ with a fixed charging capacity of 250 mAh g^−1^, as shown in Figure 3b. The room temperature ionic liquid Al-Cl_2_ battery shows rapid capacity decay. In contrast, the Al-Cl_2_ battery using the molten salt maintains stable operation over 300 cycles, achieving an average coulombic efficiency of 96.56% and a reversible average discharge capacity of 241.5 mAh g^−1^. The compositional differences between ionic liquids and molten salts arise from their respective cations: ionic liquids contain EMI^+^ cations, while molten salts consist of alkali metal ions such as Li^+^, Na^+^, and K^+^. We suggest that the presence of EMI^+^ ions leads to irreversible redox reactions involving Cl_2_.

To clarify the effect of EMI^+^ ions on Cl_2_, we calculated the adsorption energy of chlorine molecules on EMIC, as shown in Figure 3c and Figure S6a. Our results reveal that the adsorption energy of EMIC for chlorine (−0.942 eV) is significantly lower than that of bare graphene (−0.213 eV) (Figure S6b), suggesting a greater likelihood of chlorine molecules adsorbing onto EMI⁺ ions. To further elucidate the effect of EMI^+^ on the irreversible cycling of chlorine, we conducted ab initio molecular dynamics (AIMD) simulations to model solvation environments involving Cl_2_, Al, Cl^−^, and cations (alkali metal ions Na, K, Li for molten salt or EMI^+^ for ionic liquids). Molecular simulation snapshots of the molten salt system show that chlorine molecules are partially concentrated near the graphene layers (Figure 3d), whereas in the ionic liquid, chlorine does not adsorb near these layers (Figure 3e). This observation is further corroborated by the peaks in the density curves illustrating the interactions of chlorine gas with other species (Figure 3f,g). EMI^+^ ions may form bonds with Cl_2_, leading to the dispersion of chlorine gas within the electrolyte rather than adsorption near the graphene layers. This implies that molten salt electrolytes are a promising option for facilitating the reversible cycling of chlorine gas. Additionally, chlorine can form C-Cl π bonds with the carbon atoms in the EMI^+^ ring, leading to additional substitution reactions [53,54]. We performed nuclear magnetic resonance (NMR) tests on the electrolyte after several cycles, and the H spectra clearly revealed the presence of impurity peaks. This observation indicates that EMI^+^ ions participate in side reactions during the oxidation process, including hydrogen chlorination on the EMI^+^ ring and decomposition of EMI^+^ (Figure S7) [53,55]. This further underscores the instability of chlorine gas in ionic liquids.

**Figure 4:**
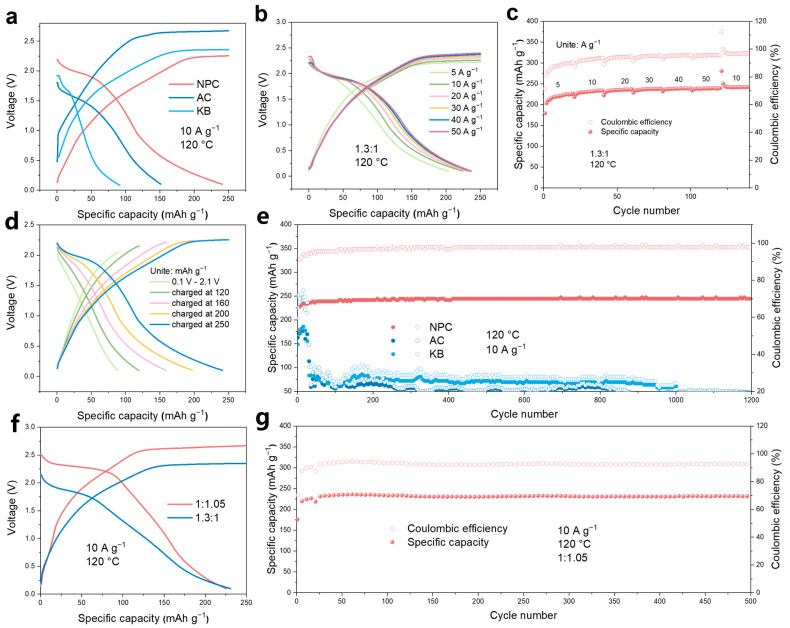
Electrochemical performances of the molten salt Al−Cl_2_ battery at 120 °C. (**a**) GCD curves of the Al−Cl_2_ battery with NPC, KB and AC. (**b**) Voltage profiles with a charge capacity of 250 mAh g^−1^ at various current rates from 5 to 50 A g^−1^. (**c**) Rate capacities at various current rates from 5 to 50 A g^−1^. (**d**) Voltage profiles of the Al−Cl_2_ battery with NPC at a current density of 10 A g^−1^ with specific capacities from 120 to 250 mAh g^−1^. (**e**) Cycling performance at a current density of 10 A g^−1^ with a charge capacity of 250 mAh g^−1^ for NPC, KB and AC. (**f**) GCD curves of the Al−Cl_2_ battery with acidic and alkaline electrolytes. (**g**) Cycling performance at a current density of 10 A g^−1^ with a charge capacity of 250 mAh g^−1^ for alkaline electrolytes.

We assessed the electrochemical performance of the Al-Cl_2_ battery through a series of electrochemical tests. We compared the GCD curves of NPC, KB, and commercial activated carbon (AC). NPC exhibits smaller overpotentials (260 mV) and higher coulombic efficiency (96.4%) compared to AC (>1000 mV, 60.5%) and KB (~500 mV, 36.7%) (Figure 4a). The NPC was assembled into a battery for cyclic voltammetry (CV) testing, revealing distinct oxidation peaks for chlorine gas evolution and reduction peaks around 2 V vs. Al^3+^/Al (Figure S8). As illustrated in Figure 4d, we examined the GCD curves of the aluminum-chlorine battery using NPC at a current of 10 A g^−1^ with varying charged specific capacities. When the voltage range was set to 0–2 V, no significant plateaus were observed. However, as the charge capacity increased from 120 mAh g^−1^ to 250 mAh g^−1^, clear discharge plateaus emerged. The coulombic efficiency exhibited only a minor decline (Figure S9), while the discharge capacity was more than double that observed in the voltage range of 0.1 to 2 V. The Al-Cl_2_ battery demonstrated excellent cycling stability and high coulombic efficiency, mainly due to the strong adsorption of chlorine gas and the abundant pore volume of the NPC.

We fixed the charge capacity at 250 mAh g^−1^ to evaluate the rate performance of the assembled Al-Cl_2_ battery using NPC. As displayed in Figure 4b, the battery demonstrates outstanding rate performance and good cycling stability across current densities ranging from 5 to 50 A g^−1^, with only a slight decrease in the associated discharge plateau. The reversible capacities attained at current densities of 5, 10, 20, 30, 40, and 50 A g^−1^ were 226.5, 233.6, 236.8, 237.7, 238.8, and 239.2 mAh g^−1^, respectively (Figure 4c). The coulombic efficiency increased with higher current densities, reaching 90.5% at 5 A g^−1^ and 95.7% at 50 A g^−1^, aligning with previously observed trends [50]. We evaluated the cycling stability of NPC, KB, and AC (Figure 4e). At a current density of 10 A g^−1^ and a charge capacity of 250 mAh g^−1^, the Al-Cl_2_ battery using AC and KB as cathodes could only support a limited number of cycles, showing a rapid decrease in capacity. In contrast, the NPC enabled stable cycling for over 1200 cycles, achieving an average coulombic efficiency of 97.3% and an average reversible discharge capacity of 243.3 mAh g^−1^ at 120 °C.

Furthermore, the Lewis acidity of the molten salt is influenced by the ratio of AlCl_3_ to alkali metal chlorides. To examine how the acidity and alkalinity of the molten salt affect the performance of the Al-Cl_2_ battery, we adjusted the ratio of AlCl_3_ to alkali metal chlorides to 1:1.05, resulting in the formation of the basic molten salt electrolyte. According to the GCD curves (Figure 4f), the Al-Cl_2_ battery using alkaline electrolytes, compared to one with acidic electrolytes, exhibit not only a higher oxidation plateau (~2.5 V) but also a higher discharge plateau (~2.3 V) and discharge specific energy (~310 Wh kg^−1^, calculated based on the cathode material). This is mainly due to changes in the Lewis acid-base characteristics of the electrolyte affecting the ion coordination environment, where the AlCl_4_^−^/Cl_2_ redox pair in the original acidic electrolyte shifts to Cl⁻/Cl_2_ [56,57]. According to the Figure 4g, under the conditions of a current of 10 A g^−1^ and a temperature of 120 °C, the alkaline electrolyte can maintain stable cycling for 500 times, with an average coulombic efficiency of 92.14% and an average discharge specific capacity of 230.4 mAh g^−1^. The alkaline electrolyte also demonstrates excellent rate performance at current densities ranging from 5 to 50 A g^−1^ (Figure S10). The reversible capacities achieved at currents of 5, 10, 20, 30, 40, and 50 A g^−1^ were 218.2, 230.3, 235.8, 237.6, 238.8, and 238.9 mAh g^−1^, respectively.

Among halogen elements, bromine (Br) and chlorine (Cl) exhibit comparable properties. To explore the reversibility of the Br redox reaction in molten salt, we replaced 20% of NaCl in the electrolyte with NaBr to assess if the Br_2_/Br^−^ couple can undergo reversible cycling in this system. As shown in Figure S11a, a distinct discharge plateau appears at a current density of 10 A g^−1^ and a temperature of 100 °C, with the charging and discharging levels notably lower than the potential of the Cl_2_/Cl^−^ couple. This indicates that reversible cycling of Br is also feasible within the molten salt system, achieving stable cycling for over 400 cycles at a current density of 10 A g^−1^ (Figure S11b).

**Figure 5:**
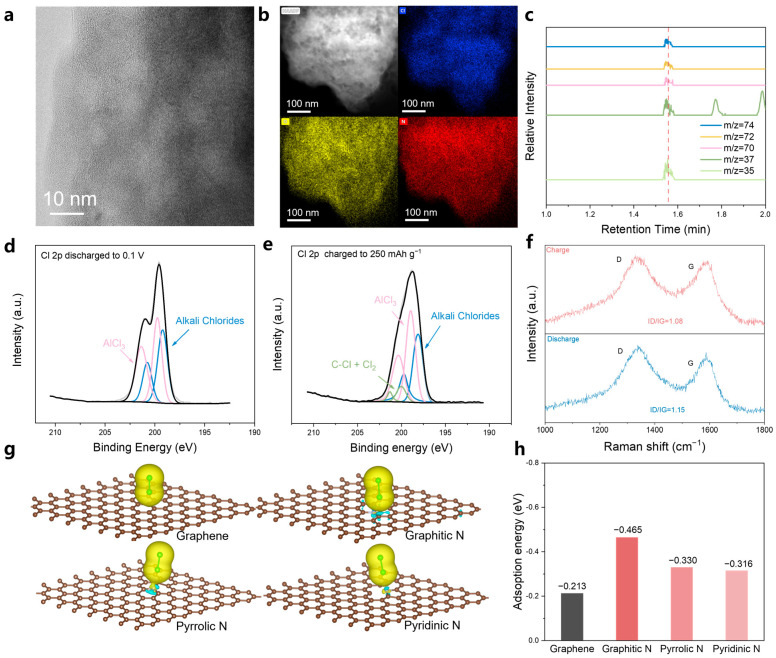
Understandings of the reaction mechanism of the molten salt Al-Cl_2_ battery. (**a**,**b**) TEM images for the NPC cathode after cycling. (**c**) EDS mapping images for NPC cathode after the battery was charged to 250 mAh g^−1^. (**d**) Cl 2p XPS spectrum of NPC electrode after the battery was discharged to 0.1 V (**e**) Cl 2p XPS spectrum of NPC electrode after the battery was charged to 250 mAh g^−1^. (**f**) Mass-spectrometry analysis of NPC electrodes with charged at 250 mAh g^−1^. (**g**) Charge density difference of the optimized sites for Cl_2_ adsorption on N-doped carbons and (**h**) the corresponding adsorption energies.

To better understand the operation mechanism of the Al-Cl_2_ battery, we performed detailed characterizations of the NPC cathode throughout the charge and discharge processes. As illustrated in Figure 5a and Figure S12, the NPC retained its porous structure after cycling, demonstrating its stability. Energy-dispersive X-ray spectroscopy (EDS) showed substantial chlorine signals in both the fully charged and discharged states, attributed to residual solid electrolytes (AlCl_3_, NaCl, KCl, and LiCl) present within the porous carbon (Figure 5b and Figure S13). We carried out gas chromatography-mass spectrometry (GC-MS) tests on the NPC charged to 250 mAh g^−1^. The NPC electrode was placed in a sealed container within a glove box, heated to 80 °C, and the gases collected from this container were analyzed using mass spectrometry. We identified the characteristic ion fragments of Cl_2_ (M/Z = 70, 72, 35, 37, 74) and observed that the peak for these fragments occurred at a retention time of 1.55 min (Figure 5c). Based on these results, we conclude that under the current testing conditions, the retention time for chlorine gas is 1.55 min, confirming the presence of Cl_2_. We also conducted ex situ X-ray photoelectron spectroscopy (XPS) analysis on the NPC cathode, measuring it in different states, specifically at a charging capacity of 250 mAh g^−1^ and discharged to 0.1 V. The spectra obtained from the discharged cathode at 0.1 V could be deconvoluted into characteristic peaks corresponding to alkali metal salts (Cl 2p3/2 at ~199.6 eV) and AlCl_3_ (Cl 2p3/2 at 201.1 eV), representing residual electrolyte components (Figure 5d). When charged to 250 mAh g^−1^, alongside the electrolyte components, distinct characteristic peaks for C-Cl and Cl_2_ (Cl 2p3/2 at 200.02 eV) were detected (Figure 5e), aligning with earlier reports [58,59]. We measured the Raman spectra of the nitrogen-doped porous carbon (NPC) under different states (Figure 5f). The initial I_D_/I_G_ ratio of the NPC is 1.14 (Figure 2b), which decreased to 1.08 when it was in the charged state and returned to 1.15 after discharging. This is attributed to the decrease in disorder degree of the NPC after chlorine adsorption, followed by recovery after discharging. These findings suggest that chlorine gas is generated and participates in the electrode reactions.

The above findings indicate that nitrogen-doped structures on NPC serve as favorable adsorption sites for Cl_2_. To further elucidate the impact of nitrogen-doped structures on Cl_2_ adsorption, we conducted DFT calculations to assess the adsorption energies of Cl_2_ on graphene and various nitrogen-doped structures, including Graphitic N, Pyrrolic N, and Pyridinic N. Charge density difference analysis (Figure 5g) through DFT calculations indicates that the strong dipole force at the center of the doped N atoms allows N atoms to serve as favorable sites for Cl_2_ adsorption. Moreover, compared to the vertical adsorption configuration induced by bare van der Waals forces on graphene, Cl_2_ tends to adsorb at the N sites due to the strong interactions induced by both dipole and van der Waals forces. As demonstrated in Figure 5h, the adsorption energies for Graphitic N (−0.465 eV), Pyridinic N (−0.330 eV), and Pyrrolic N (−0.316 eV) are all lower than that of bare graphene (−0.213 eV). The lowest adsorption energy of Graphitic N with Cl_2_ demonstrates its strongest adsorption capability. This indicates that nitrogen-doped structures significantly enhance the Cl_2_ adsorption capability of the porous carbon cathode, contributing to high-rate performance and good cycling stability.

**Figure 6:**
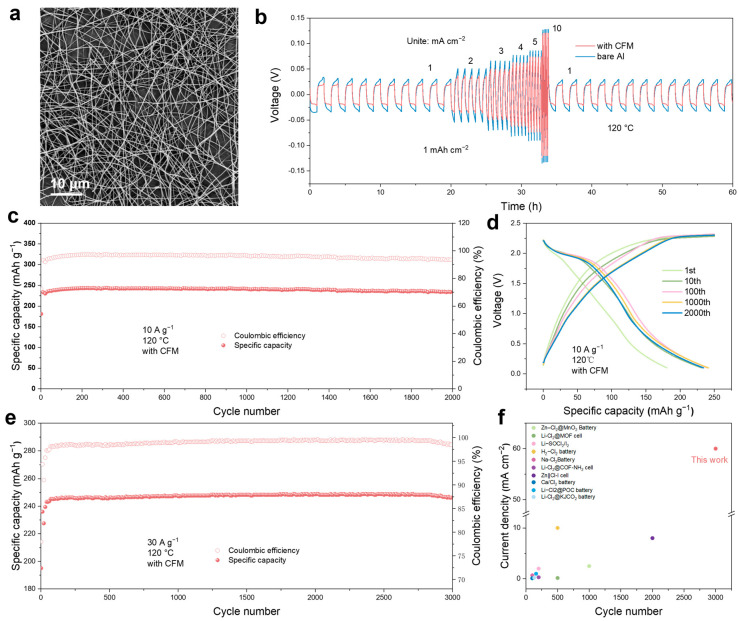
Electrochemical performances of the molten salt Al-Cl_2_ battery using an anode protection layer. (**a**) SEM images of the CFM. (**b**) Rate performance of Al||Al and Al@CFM||CFM@Al symmetric cells at various current densities from 1 to 10 mA cm^−2^ with a constant striping/plating capacity of 1 mAh cm^−2^. (**c**,**d**) Cycling performance and voltage profiles of the Al−Cl_2_ battery with the CFM at 120 °C at a current density of 10 A g^−1^ and a fixed charge capacity of 250 mAh g^−1^. (**e**) Cycling performance of the Al−Cl_2_ battery with the CFM at a current density of 30 A g^−1^. (**f**) Comparison of the cycle performance and rate performance of our Al-Cl_2_ battery and recently reported chlorine-based conversion reaction batteries [34,35,37,41,43,50,59,60,61].

Continuous cycling at high current densities leads to significant issues with aluminum dendrite formation on the anode side (Figure S14). To improve the cycle life of the Al-Cl_2_ battery, we developed a carbon fiber membrane (CFM) through electrospinning and sintering. This membrane was applied to the anode side to promote uniform ion deposition and reduce the formation of aluminum dendrites. SEM and TEM images (Figure 6a and Figure S15a) show that the carbon nanofibers have a diameter of approximately 200 nm. EDS analysis indicates a uniform distribution of carbon (C), nitrogen (N), and oxygen (O) elements (Figure S15b). To elucidate the effect of CFM coating on the reversible Al plating/stripping, we assembled an Al@CFM-based symmetric cell for constant current charge/discharge cycling tests at 1 mA cm^−2^ and 1 mAh cm^−2^. As depicted in Figure S16, the Al@CFM-based symmetric battery can stably cycle for over 600 h, while the symmetric battery without CFM exhibits significant voltage fluctuations and experiences short circuits. Under conditions of 5 mA cm^−2^ and 1 mAh cm^−2^, the symmetric cell without the CFM quickly short-circuited (Figure S17b). In contrast, the symmetric battery with the CFM maintained stable cycling for over 140 h, maintaining a low voltage polarization of about 200 mV (Figure S17a). To assess the effect of the CFM on Al deposition behavior, we examined the Al anode after different numbers of cycles. SEM images revealed a smooth pristine aluminum surface (Figure S18). Figure S19 illustrates that the Al anode with the CFM showed a flat deposition morphology after cycling tests, whereas significant dendrite growth was observed on the Al anode without the CFM. Figure 6b shows the rate performance of the Al@CFM||Al@CFM and the Al||Al symmetric battery at different current densities ranging from 1 to 10 mA cm^−2^ with a fixed striping/plating capacity of 1.0 mAh cm^−2^. The cell equipped with CFM displayed voltage polarizations of 44, 70, 103, 126, 147, and 240 mV at current densities of 1, 2, 3, 4, 5, and 10 mA cm^−2^, respectively. These values are lower than the corresponding voltage polarizations of bare aluminum, indicating that the CFM aids in the desolvation of Al^+3^, leading to reduced voltage polarization during deposition and dissolution reactions, thus enhancing cycling stability.

We also assembled a rechargeable molten salt Al-Cl_2_ battery with the CFM protection layer. As shown in Figure 6c, the battery achieved stable cycling for over 2000 cycles at a current density of 10 A g^−1^. The average coulombic efficiency and average discharge specific capacity of this Al-Cl_2_ battery are 95.75% and 239.4 mAh g^−1^, respectively. Figure 6d illustrates the GCD curve, which indicates good cycling stability, featuring a clear discharge plateau even after 2000 cycles at high currents. To further illustrate the impact of CFM on the cycling life of the Al-Cl_2_ battery, we conducted charge–discharge cycling tests at a current of 30 A g^−1^, as shown in the Figure 6e. The battery achieved over 3000 stable cycles. The average coulombic efficiency and average discharge specific capacity of the Al-Cl_2_ battery with the CFM protection layer are 98.96% and 247.4 mAh g^−1^, respectively. This suggests that the incorporation of CFM significantly enhances the cycling life of the Al-Cl_2_ battery. Electrochemical impedance spectroscopy (EIS) measurements were performed on Al-Cl_2_ batteries with the CFM, to assess their electrochemical kinetics (Figure S20). The charge transfer resistance of Al@CFM||NPC is only 97.96 Ω, which indicates rapid charge transfer in Al@CFM||NPC. To further assess the advantages of the Al-Cl_2_ battery, we compared our molten-salt Al-Cl_2_ battery with previously reported metal-chlorine batteries. As illustrated in Figure 6f and Table S2, the Al-Cl_2_ battery demonstrates excellent cycling stability and rate performance when compared to other chlorine-based conversion reaction batteries [60,61].

## 4. Conclusions

In summary, we have demonstrated a molten salt aluminum-chlorine battery that can operate stably at an elevated temperature of 120 °C, demonstrating strong rate performance and an extended cycling lifespan. Both experimental and theoretical analyses suggest that the strong adsorption of Cl_2_ by EMI^+^ ions in ionic liquids leads to an irreversible chlorine conversion reaction. The use of molten salt electrolytes promotes reversible oxidation-reduction reactions involving chlorine in aluminum-ion batteries, thus expanding the electrochemical window. Furthermore, the nitrogen-doped carbon structure improves the cathode’s ability to adsorb Cl_2_, leading to increased coulombic efficiency and reduced overpotential. The battery using this carbon cathode achieves an average discharge capacity of 243.3 mAh g^−1^ at a rate of 10 A g^−1^ and a fixed charging capacity of 250 mAh g^−1^, while maintaining stable cycling for over 1200 cycles. A series of electrochemical and characterization tests confirmed the reversible conversion of chlorine gas during cycling. To further enhance the cycle life, we applied carbon nanofiber membranes to the anode, which effectively suppresses dendrite formation. This modification allows for stable cycling for over 3000 cycles at a current of 30 A g^−1^. This work paves the way for the rational design of cost-effective, high-power metal-chlorine batteries.

## Data Availability

The original contributions presented in this study are included in the article/Supplementary Material. Further inquiries can be directed to the corresponding authors.

## Supplementary Materials

The following supporting information can be downloaded at: https://www.mdpi.com/article/10.3390/ma18081868/s1, Figure S1: XRD and Raman images of NPC, KB@COF and KB.; Figure S2: XPS spectra and C 1s XPS spectra of NPC; Figure S3: Nitrogen adsorption/desorption isotherms and relative pore size distribution of KB; Figure S4: Relative pore size distribution of the NPC; Figure S5. SEM image of the NPC; Figure S6: (a) Simulated optimized geometric structures of Cl_2_ with EMIC. (b) Charge density difference of the optimized sites for Cl2 adsorption on Graphene and the corresponding adsorption energies; Figure S7: ^1^H NMR spectra of Original ionic liquid and ionic liquid after cycling. Figure S8: CV curves of the Al_2_−Cl_2_ battery with NPC at a scan rate of 1 mV s^−1^. Figure S9: (a) Cycling performance tests at different charging capacities. (b) Coulombic efficiency of Al-Cl_2_ at different charge capacities. Figure S10: (a) Rate capacities at various current rates from 5 to 50 A g^−1^. (b) Voltage profiles of the Al−Cl_2_ battery with alkaline electrolytes at a current density of 10 A g^−1^ with charge specific capacities 250 mAh g^−1^; Figure S11: (a) Voltage profiles of the Al−Cl_2_ battery with the electrolyte replaced by 20% NaBr at a current density of 10 A g^−1^ with charge specific capacities 200 mAh g^−1^. (b) Cycling performance of the Al−Cl_2_ battery with the electrolyte replaced by 20% NaBr at a current density of 10 A g^−1^ with charge specific capacities 250 mAh g^−1^; Figure S12: TEM image of the NPC cathode after cycling; Figure S13. EDS images of the NPC when discharged to 0.1 V; Figure S14: SEM images of the aluminum anode after cycling; Figure S15: (a) TEM images of CFM. (b) EDS mapping images of elements for CFM; Figure S16: Charge/discharge cycling curves of Al || Al and Al@CFM || CFM@Al under a condition 1 mA cm^−2^, 1 mAh cm^−2^ and 100 °C; Figure S17: (a) Charge/discharge cycling curves of Al@NFM||NFM@Al under a condition 5 mA cm^−2^ and 1 mAh cm^−2^. (b)Charge/discharge cycling curves of Al || Al under a condition 5 mA cm^−2^ and 1 mAh cm^−2^; Figure S18: SEM image of pristine Al foil; Figure S19: SEM images of Al anodes without CFM (a,b) and with CFM (c,d) tested under different conditions; Figure S20. Nyquist plots of the Al-Cl_2_ battery with CFM; Figure S21. SEM micrograph of the NPC electrode; Figure S22: EDS of the NPC electrode; Figure S23: Cross-sectional SEM micrograph of the NPC electrode; Table S1: The C, N, O, H elemental content of the KB@COF and NPC sample; Table S2: Comparison of the cycle performance and rate performance of our Al-Cl2 battery and recently reported chlorine-based conversion reaction batteries. Refs [62,63,64,65,66,67,68,69,70] are included in the Supplementary Material.
