# Supplementary material for: Ultrafast Rechargeable Aluminum-Chlorine Batteries Enabled by a Confined Chlorine Conversion Chemistry in Molten Salts
Source: Materials (Basel). 2025 Apr 18;18(8):1868. doi: 10.3390/ma18081868 (PMC12028977; doi:10.3390/ma18081868)
Supplement: Supplementary file 1 [file materials-18-01868-s001.zip › materials-3566791-supplementary.pdf]

Supporting Information

Ultrafast rechargeable aluminum-chlorine batteries enabled by a confined chlorine conversion chemistry in molten salts

Junling Huang, Linhan Xu, Yu Wang, Xiaolin Wu, Meng Zhang, Hao Zhang, Xin Tong, Changyuan Guo, Kang Han, Jianwei Li, Jiashen Meng and Xuanpeng Wang**

Materials. All reagents and materials used in this work are commercially available without further purification: glass-fiber separator (GF-D, Whatman), Commercial activated carbon (AC, Fuzhou Yihuan Carbon., Ltd.), Sodium chloride (NaCl, Shanghai Aladdin), Potassium chloride (KCl, Shanghai Aladdin), Lithium chloride (LiCl, Shanghai Aladdin), Aluminium chloride ($AlCl_3$, Acros), Ketjenblack (KB, Lion), 1-ethyl-3-methylimidazoliumchloride (EMIC, Sigma-Aldrich), Polytetrafluoroethylene (PTFE, Shanghai Aladdin), Isopropanol (Shanghai Aladdin), Molybdenum (Mo, Shanghai Aladdin), Aluminum flake (Al flake, Shanghai Aladdin), Zinc chloride ($ZnCl_2$, Macklin), *N*, *N*-Dimethylformamide (DMF, Shanghai Aladdin), polyacrylonitrile (PAN, average M_w = 149,000 ~ 151,000, Shanghai Aladdin).

Preparation of NPC. First, KB, NaCl–KCl–$ZnCl_2$, and DCB are mixed uniformly in a mass ratio of 1:1:1, then the mixture is placed in a dry quartz tube, which is evacuated and sealed. It is heat-treated at a temperature of 300°C for 48 hours (with a heating rate of 5°C/min and furnace cooling), resulting in KB@COF. KB@COF was transferred into a crucible, and then heated at 800 °C for 3 h under argon atmosphere with a heating rate of 5°C min^{-1} to obtain NPC material.

Preparation of electrodes. NPC was mixing with PTFE with a mass ratio of 4:1 and ground in an agate mortar. Then, the mixture is cut into rectangles. Active mass is 2 - 4 mg cm^{-2} based on the rectangular area.

Preparation of electrolytes. All the operations below were performed inside the argon-filled glovebox (condition: O_2 <0.01 ppm, H_2O < 0.01 ppm). For the preparation of acid molten salt electrolytes, anhydrous $AlCl_3$, NaCl, LiCl, and KCl with an optimized molar ratio of 1.3:0.43:0.42:0.15 were added into a glass weighing flask. For alkaline molten salt electrolytes, the ratio is 1:0.452:0.441:0.158.The flask was sealed and heated to 160 °C in an oven for 12 h, yielding a homogeneous clear liquid. After cooling down to room temperature, the solid mixture was grinded thoroughly to obtain the powder electrolyte. For the preparation of the organic ionic liquid electrolyte, anhydrous $AlCl_3$ and EMIC with a mole ratio of 1.3:1 was mixed thoroughly. During the mixing process, the anhydrous $AlCl_3$ was slowly added into EMIC at room temperature under rigorous stirring, eventually forming a transparent liquid.

Preparation of Al-Cl_2 batteries. For the Al-Cl_2 battery, a house-designed Swagelok cell with a protection sheath was assembled using a commercial Al foil as the anode, a glass fiber as the separator, the quaternary chloroaluminate melt as the electrolyte and NPC (Ac, KB) as the cathode. The thickness of commercial Al foils is 0.2mm. The dosage of electrolyte in each cell is ~100mg. These cells were placed into a constant temperature chamber at 120 °C.

Preparation of CFM. 1.8 g of polyacrylonitrile (PAN) was dissolved in 22 mL of dimethylformamide (DMF). Electrospinning was performed using a syringe at an applied voltage of 10 kV. The collector distance was set to 15 cm, and aluminum foil was used as the collector. The collected fiber mat was first heated in air at a rate of 3 ℃ per minute up to 220 ℃ and held at this temperature for 1 hour. Subsequently, it was further heated under an argon atmosphere at a rate of 5 ℃ per minute up to 600 ℃ and maintained at this temperature for 1 hour, resulting in carbonized fiber mat (CFM).

Characterizations. The XRD characterization was measured using a Bruker D8 Discover X-ray diffractometer with a Cu Kα X-ray source. The Raman spectra were collected using a Renishaw INVIA micro-Raman spectroscopy system. The XPS measurement was performed using a VG MultiLab 2000 instrument. The SEM images were obtained using a JEOL JSM-7100F at an acceleration voltage of 20 kV. The Brunauer–Emmett–Teller (BET) and Barret–Joyner–Halenda (BJH) plots was performed from nitrogen adsorption isotherms collected at 77 K using a Tristar-3020 instrument. Transmission electron microscopy (TEM) and energy dispersive X-ray spectroscopy (EDS) mapping images were taken by ThermoFisher Titan Themis G2 60-300.

Electrochemical measurements. The charge-discharge test for Al-Cl_2 was measured using a Swagelok battery on a Neware test system. For the Al–Cl_2 battery, a Swagelok cell was assembled using a commercial Al foil as the anode, a glass fiber as the separator, the quaternary chloroaluminate melt as the electrolyte and NPC (KB or AC) as the cathode. The thickness of commercial Al foils is 0.2mm. The dosage of electrolyte in each cell is ~100mg. The test temperature of the battery is maintained by placing the battery in a forced air drying oven. Electrochemical impedance spectroscopy (EIS) and cyclic voltammetry (CV) tests were performed on a Bio-Logic VMP-3 electrochemical workstation.

Theoretical calculations. Our simulations employed density functional theory (DFT)[1, 2] with the Perdew−Burke−Ernzerhof (PBE) exchange−correlation functional[3] in the Vienna Ab initio Simulation Package (VASP)[4, 5]. The standard VASP projector-augmented-wave (PAW)[6] potentials were utilized to model the nuclei and frozen-core electrons of H, C, N, Al, and Cl. The corresponding self-consistently optimized valence electron configurations were $1s^1$ for H, $2s^22p^2$ for C, $2s^22p^3$ for N, $3s^23p^1$ for Al, and $3s^23p^5$ for Cl. The electronic wave functions were expanded in plane waves up to a kinetic energy cutoff of 600 eV. The Brillouin zone (BZ) integrals were computed using a Monkhorst−Pack[7] sampling scheme, employing a 1 × 1 × 1 k-point mesh for the molten salt model, which has hexagonal lattice of 19.75 Å × 19.75 Å × 19.80 Å, and a 2 × 2 × 1 k-point mesh for the ionic liquid model, with hexagonal lattice of 14.81 Å × 14.81 Å × 21.50 Å. We integrated the Brillouin zone using the Gaussian smearing method, with a smearing width of 0.05 eV. Density profiles of Cl_2 was investigated via the Ab initio molecular dynamic simulation (AIMD) calculations at 373K for at least 10 ps in the canonical ensemble, the Nose-Hoover thermostat used a 2fs time step.[8] The long-range van der Waals dispersion interactions were treated by the DFT-D4 method of Grimme[9] in the adsorption energy calculation of Cl_2. The relaxation of the lattice parameters and atomic coordinates converged when the forces were smaller than 0.01 eV/Å.

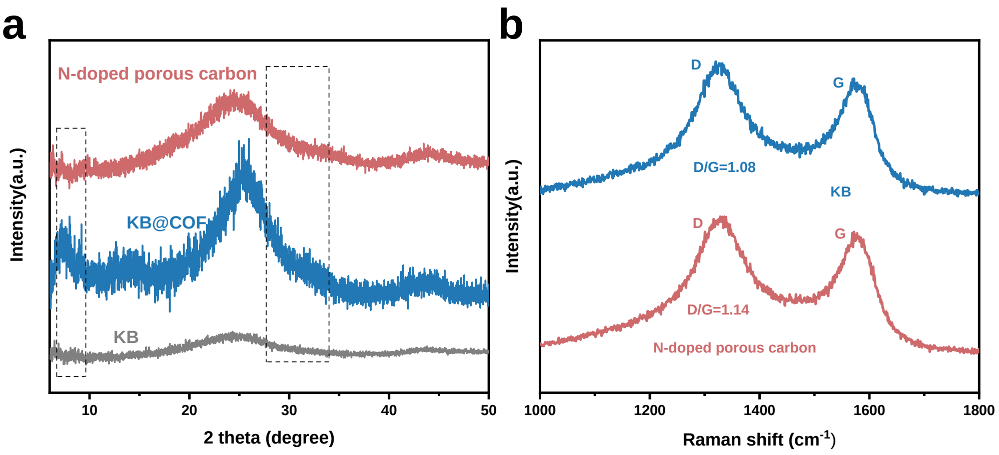

Figure S1. (a) XRD images of NPC, KB@COF and KB. (b) Raman spectroscopy of NPC and KB.

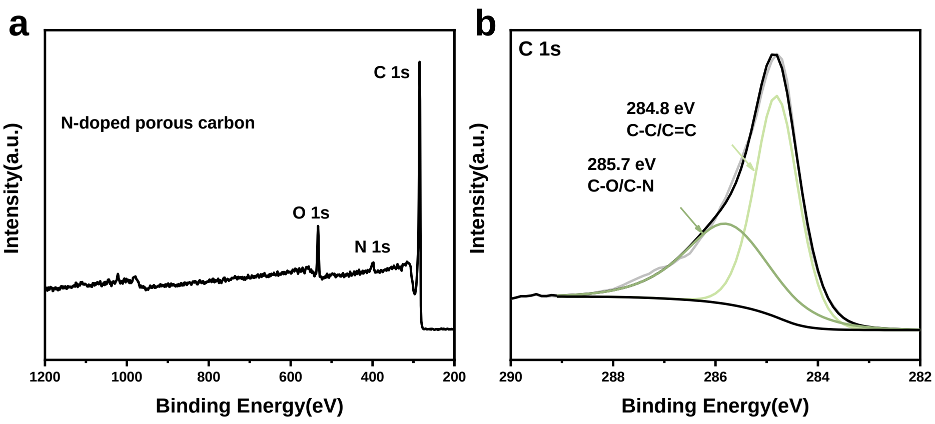

Figure S2. (a) XPS spectra of NPC. (b) C 1s XPS spectra of NPC.

Table S1. The C, N, O, H elemental content of the KB@COF and NPC sample.

	N (%)	C(%)	O(%)	H(%)
KB@COF	10.92	77.92	5.074	2.218
NPC	3.23	89.21	3.421	0.858

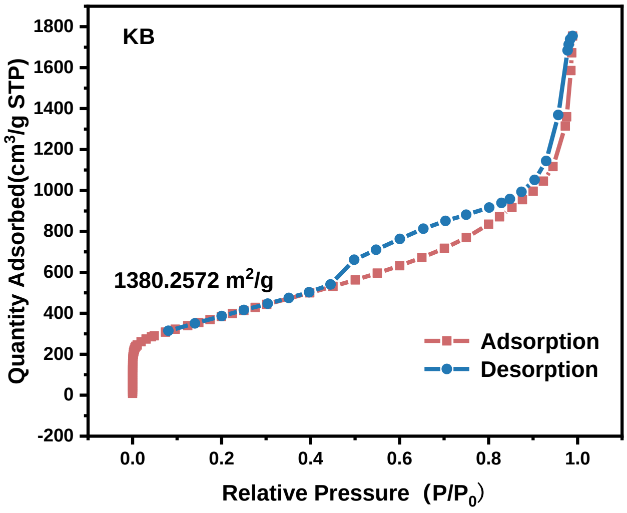

Figure S3. Nitrogen adsorption/desorption isotherms and relative pore size distribution of KB.

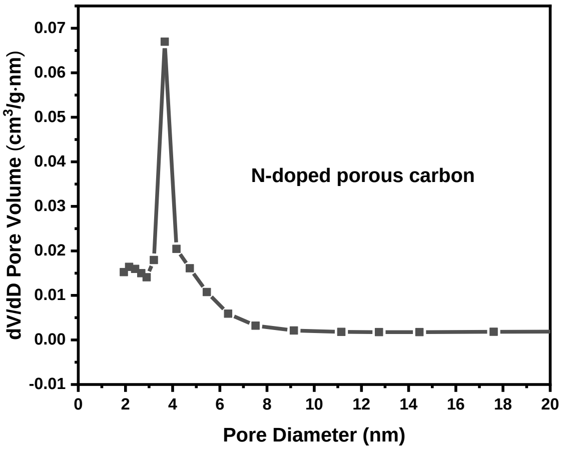

Figure S4. Relative pore size distribution of the NPC.

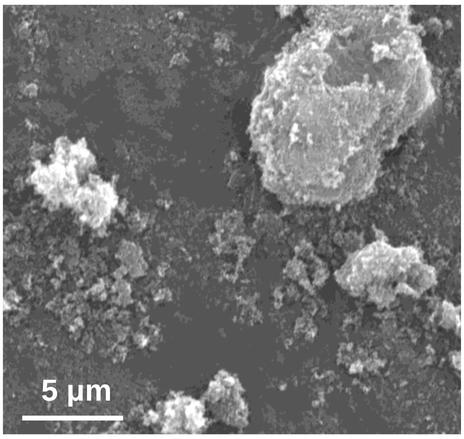

Figure S5. SEM image of the NPC.

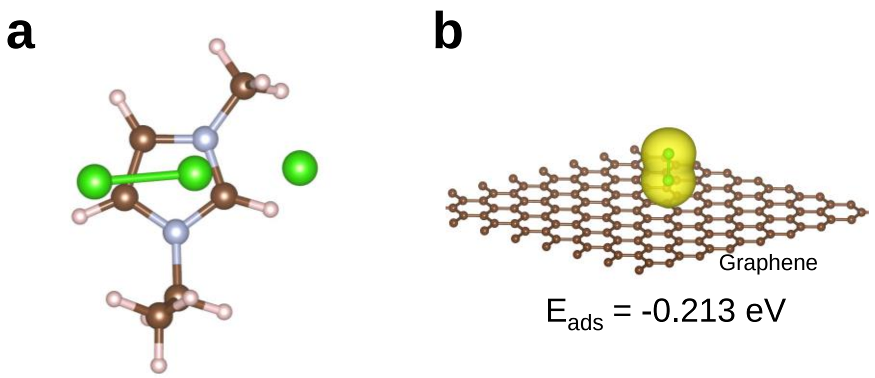

Figure S6. (a) Simulated optimized geometric structures of Cl_2 with EMIC. (b) Charge density difference of the optimized sites for Cl2 adsorption on Graphene and the corresponding adsorption energies.

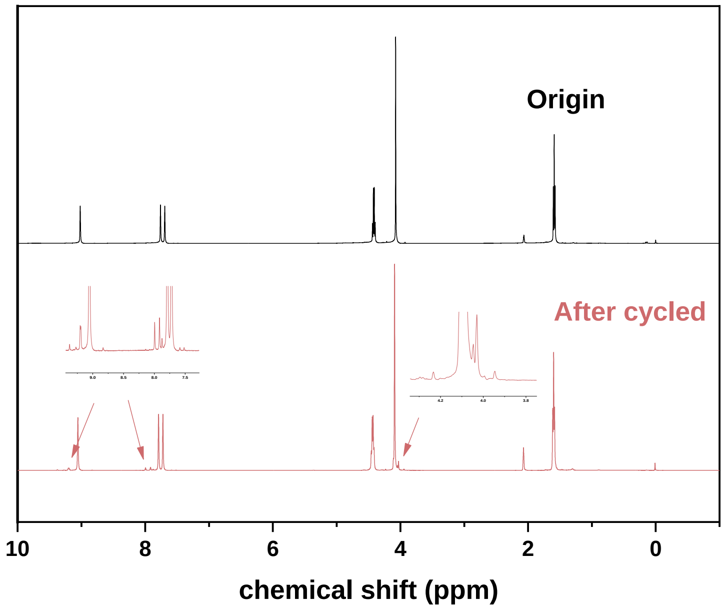

Figure S7. ^{1}H NMR spectra of Original ionic liquid and ionic liquid after cycling.

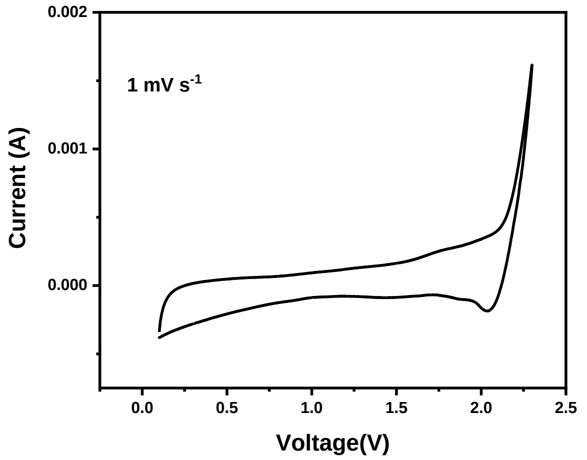

Figure S8. CV curves of the Al_2–Cl_2 battery with NPC at a scan rate of 1 mV s^{-1}.

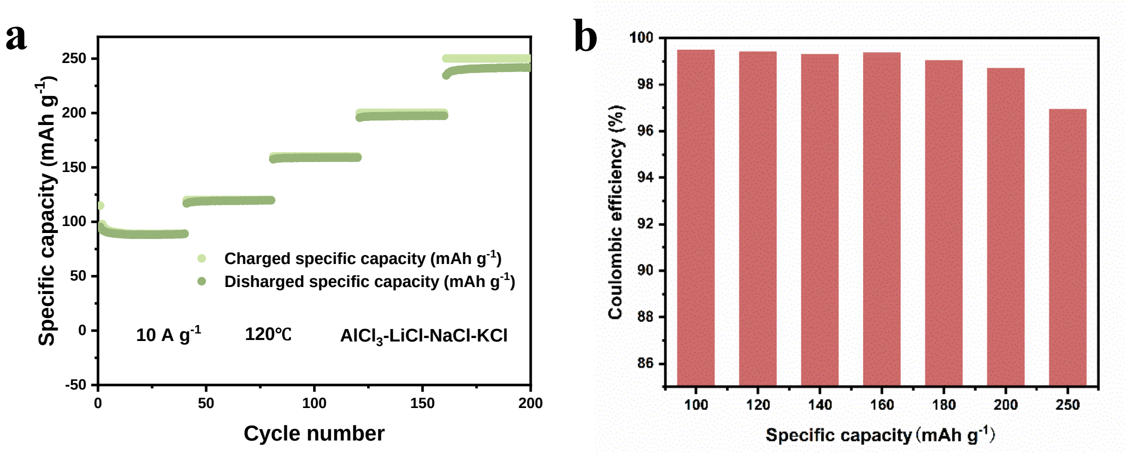

Figure S9. (a) Cycling performance tests at different charging capacities. (b) Coulombic efficiency of Al-Cl_2 at different charge capacities.

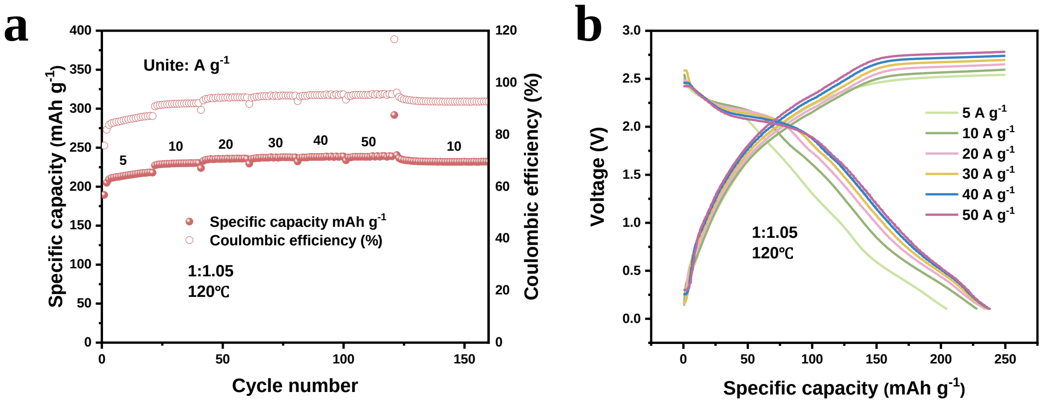

Figure S10 (a) Rate capacities at various current rates from 5 to 50 A g^{-1}. (b) Voltage profiles of the Al−Cl_2 battery with alkaline electrolytes at a current density of 10 A g^{-1} with charge specific capacities 250 mAh g^{-1}.

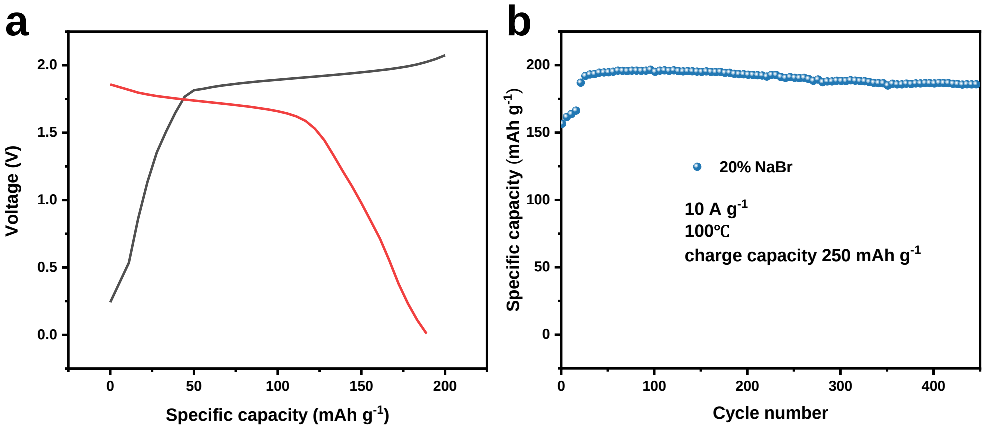

Figure S11 (a) Voltage profiles of the Al−Cl_2 battery with the electrolyte replaced by 20% NaBr at a current density of 10 A g^{-1} with charge specific capacities 200 mAh g^{-1}. (b) Cycling performance of the Al−Cl_2 battery with the electrolyte replaced by 20% NaBr at a current density of 10 A g^{-1} with charge specific capacities 250 mAh g^{-1}.

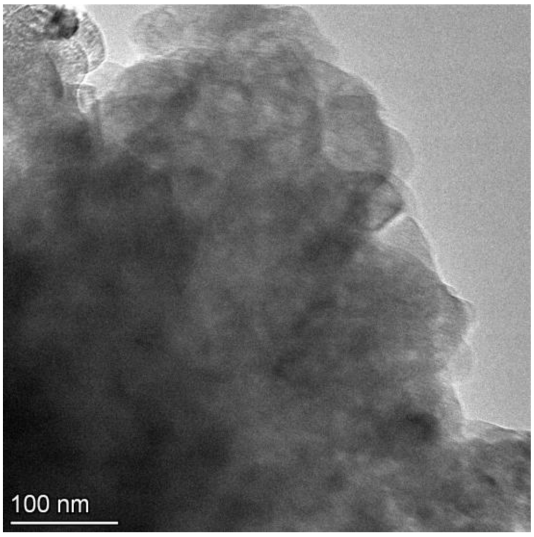

Figure S12. TEM image of the NPC cathode after cycling.

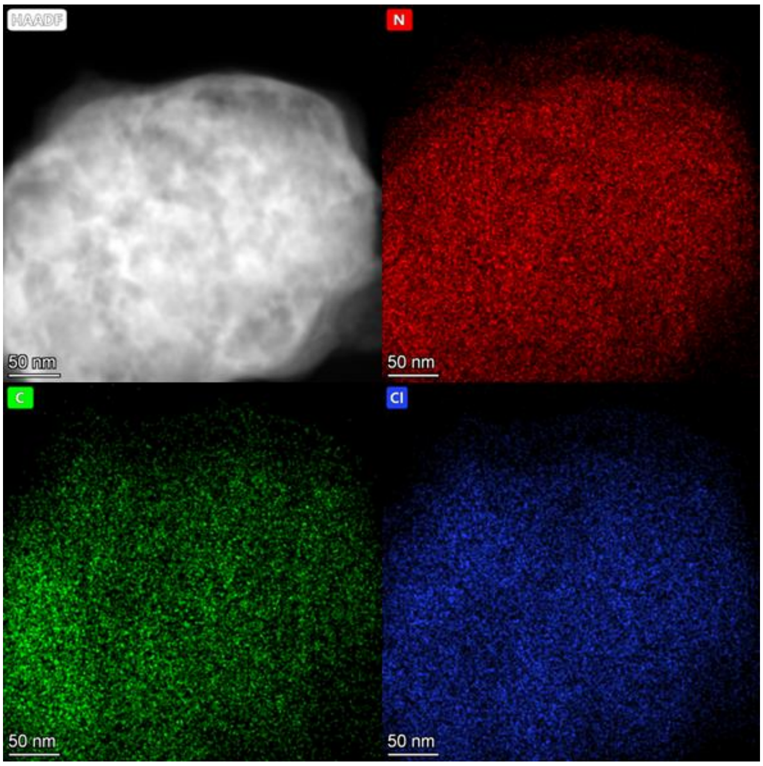

Figure S13. EDS images of the NPC when discharged to 0.1 V.

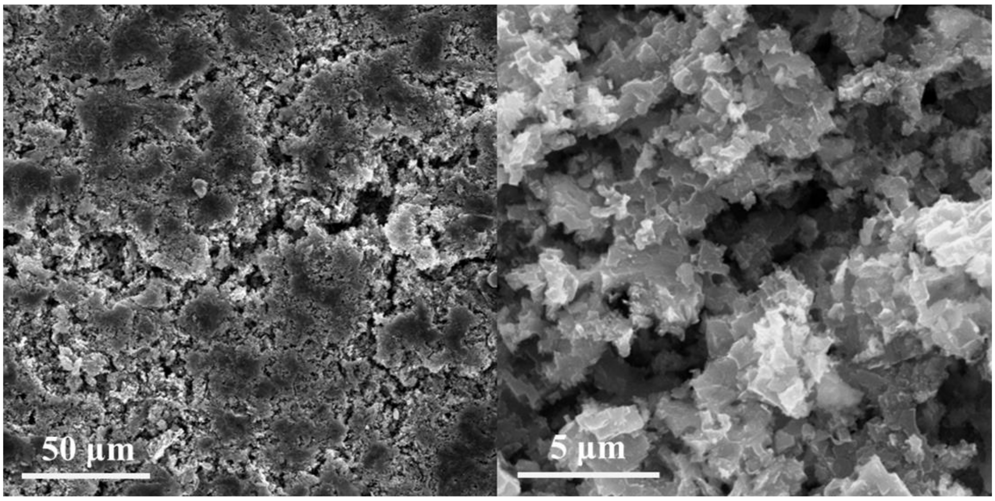

Figure S14. SEM images of the aluminum anode after cycling.

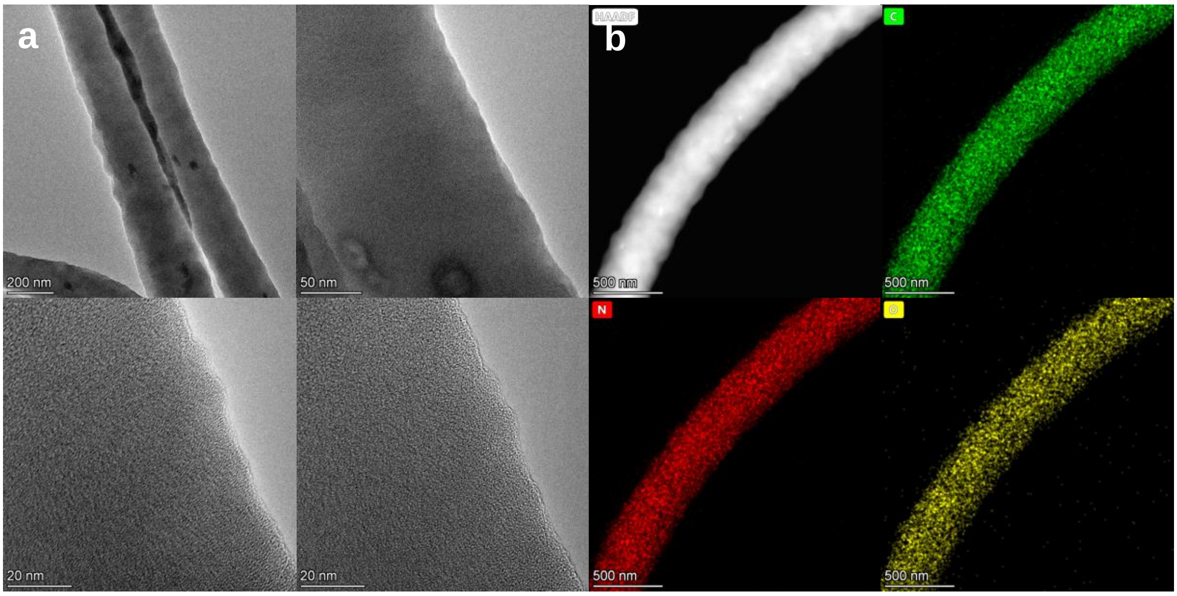

Figure S15. (a) TEM images of CFM. (b) EDS mapping images of elements for CFM.

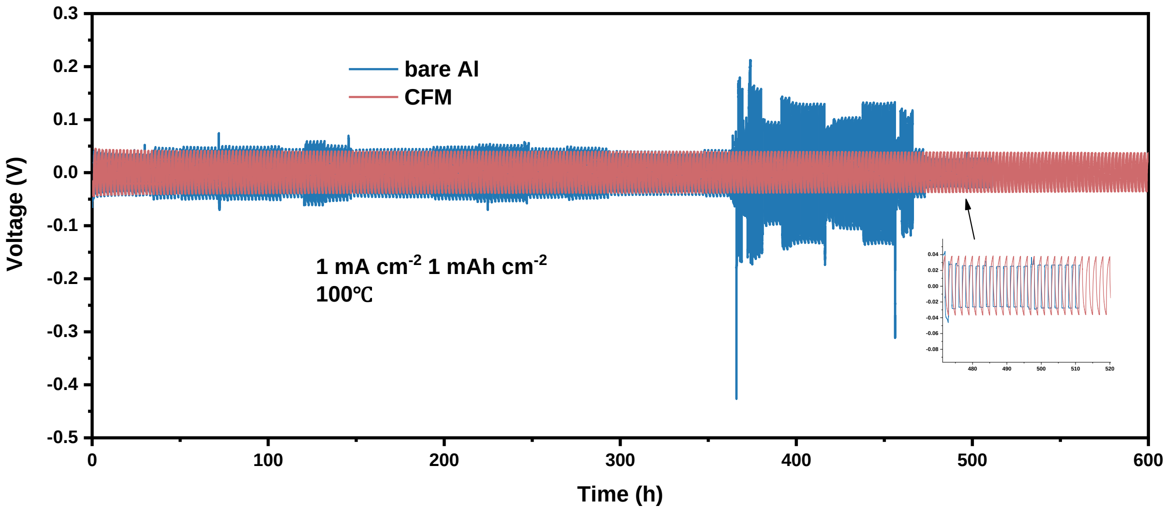

Figure S16. Charge/discharge cycling curves of Al || Al and Al@CFM || CFM@Al under a condition 1 mA cm^{-2}, 1 mAh cm^{-2} and 100 °C.

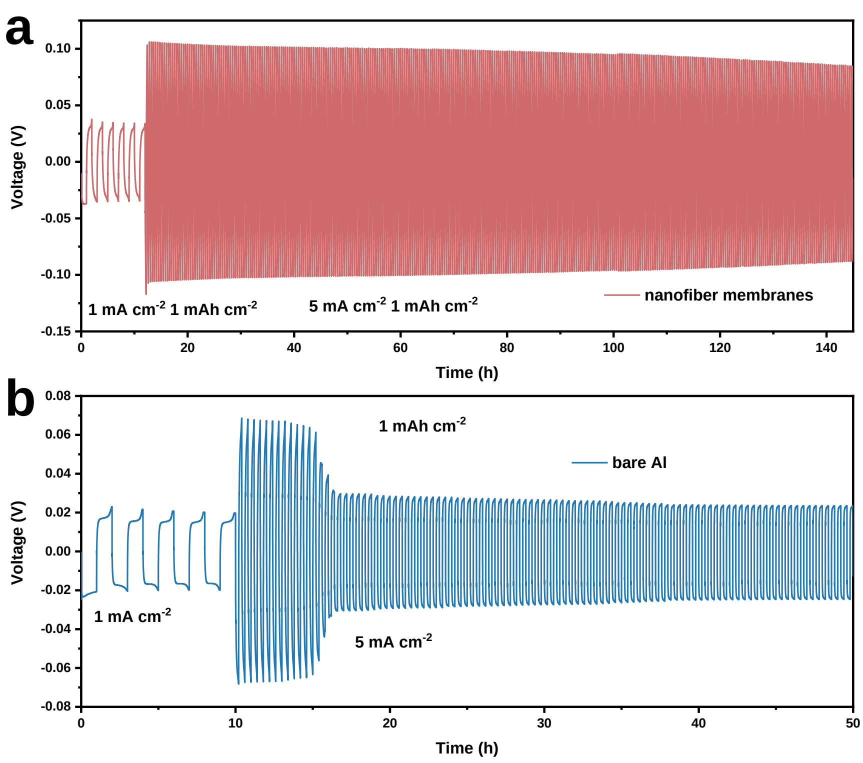

Figure S17. (a) Charge/discharge cycling curves of Al@NFM||NFM@Al under a condition 5 mA cm^{-2} and 1 mAh cm^{-2}. (b)Charge/discharge cycling curves of Al || Al under a condition 5 mA cm^{-2} and 1 mAh cm^{-2}.

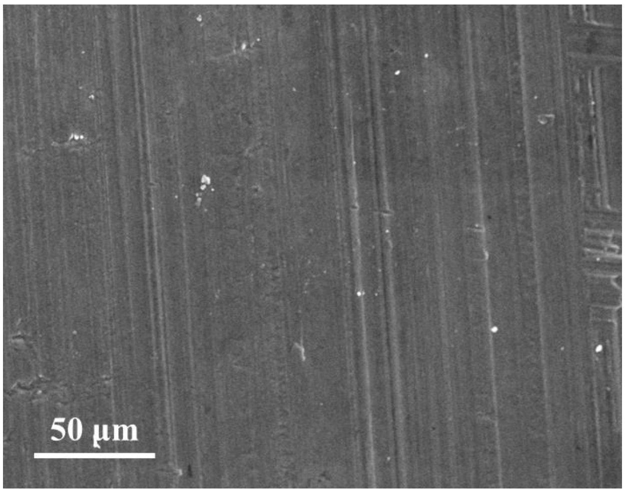

Figure S18. SEM image of pristine Al foil.

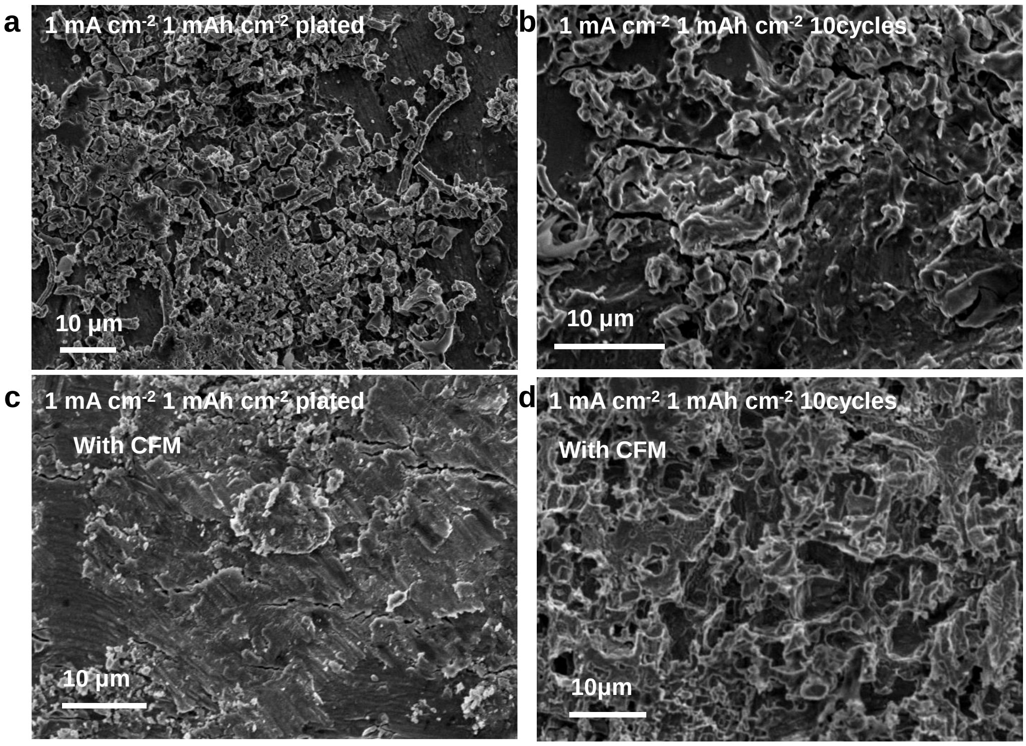

Figure S19. SEM images of Al anodes without CFM (a, b) and with CFM (c, d) tested under different conditions

Table S2. Comparison of the cycle performance and rate performance of our Al-Cl2 battery and recently reported chlorine-based conversion reaction batteries.

cycle number	current density (mA cm^{-2})	references
500	0.15	Ref. 34
200	2	Ref. 43
500	10	Ref. 50
100	0.675	Ref. 41
200	0.3	Ref. 35
2000	8	Ref. 37
100	0.1	Ref. 60
160	1	Ref. 61
130	0.2	Ref. 59

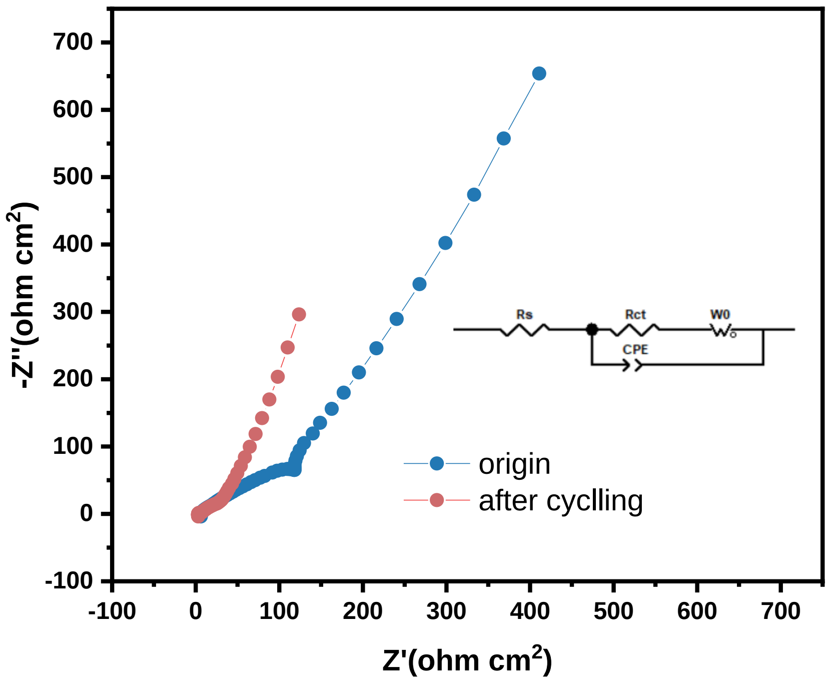

Figure S20. Nyquist plots of the Al-Cl_2 battery with CFM.

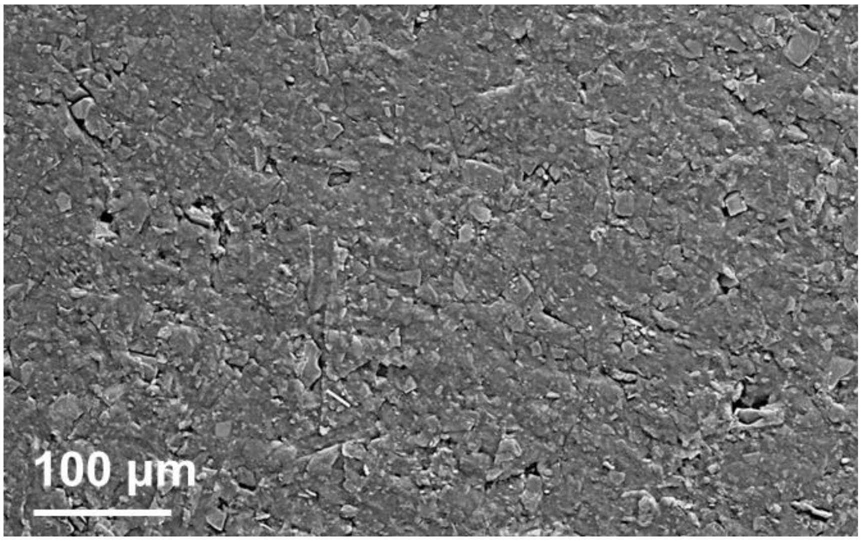

Figure S21. SEM micrograph of the NPC electrode.

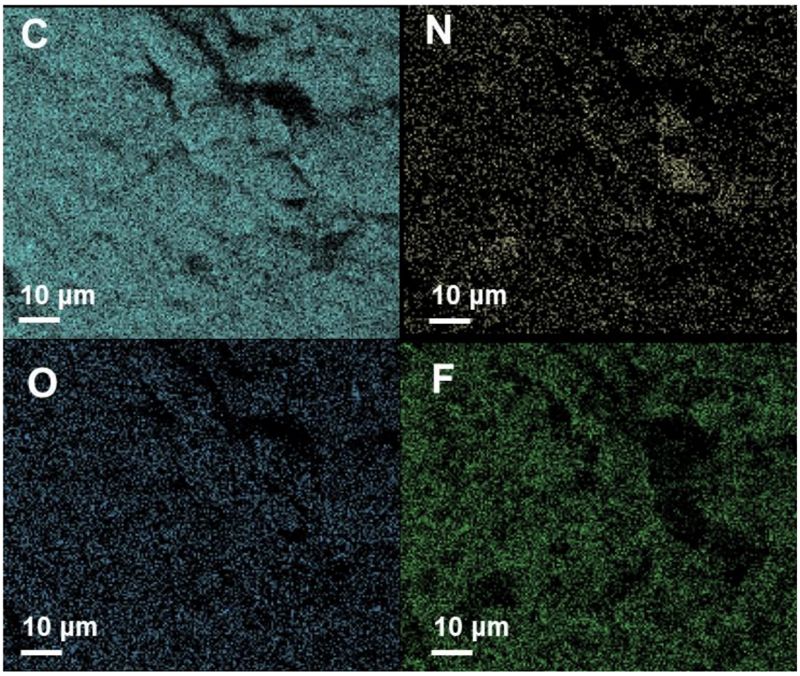

Figure S22. EDS of the NPC electrode.

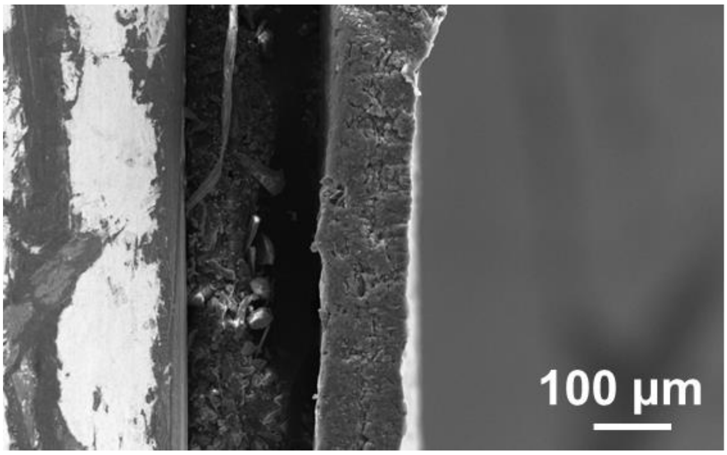

Figure S23. Cross-sectional SEM micrograph of the NPC electrode.

References

1. Hohenberg, P.; Kohn, W., Inhomogeneous Electron Gas. *Physical Review* **1964,** *136*, B864-B871.
2. Kohn, W.; Sham, L. J., Self-Consistent Equations Including Exchange and Correlation Effects. *Physical Review* **1965,** *140*, A1133-A1138.
3. Perdew, J. P.; Burke, K.; Ernzerhof, M., Generalized Gradient Approximation Made Simple. *Phys. Rev. Lett.* **1996,** *77*, 3865-3868.
4. Kresse, G.; Furthmüller, J., Efficient iterative schemes for ab initio total-energy calculations using a plane-wave basis set. *Physical Review B* **1996,** *54*, 11169-11186.
5. Kresse, G.; Furthmüller, J., Efficiency of ab-initio total energy calculations for metals and semiconductors using a plane-wave basis set. *Comput. Mater. Sci* **1996,** *6*, 15-50.
6. Blöchl, P. E., Projector augmented-wave method. *Physical Review B* **1994,** *50*, 17953-17979.
7. Monkhorst, H. J.; Pack, J. D., Special points for Brillouin-zone integrations. *Physical Review B* **1976,** *13*, 5188-5192.
8. Hoover, W. G., Canonical dynamics: Equilibrium phase-space distributions. *Physical Review A* **1985,** *31*, 1695-1697.
9. Caldeweyher, E.; Ehlert, S.; Hansen, A.; Neugebauer, H.; Spicher, S.; Bannwarth, C.; Grimme, S., A generally applicable atomic-charge dependent London dispersion correction. *The Journal of Chemical Physics* **2019,** *150*.
